# EGFR associated expression profiles vary with breast tumor subtype

**DOI:** 10.1186/1471-2164-8-258

**Published:** 2007-07-31

**Authors:** Katherine A Hoadley, Victor J Weigman, Cheng Fan, Lynda R Sawyer, Xiaping He, Melissa A Troester, Carolyn I Sartor, Thais Rieger-House, Philip S Bernard, Lisa A Carey, Charles M Perou

**Affiliations:** 1 Curriculum in Genetics and Molecular Biology, University of North Carolina at Chapel Hill, Chapel Hill, NC, USA; 2 Department of Genetics, University of North Carolina at Chapel Hill, Chapel Hill, NC, USA; 3 Lineberger Comprehensive Cancer Center, University of North Carolina at Chapel Hill, Chapel Hill, NC, USA; 4 Department of Biology, Program of in Bioinformatics and Computational Biology, University of North Carolina at Chapel Hill, Chapel Hill, NC, USA; 5 Division of Hematology/Oncology, Department of Medicine, University of North Carolina at Chapel Hill, Chapel Hill, NC, USA; 6 Department of Public Health – Biostatistics and Epidemiology Concentration, University of Massachusetts Amherst, Amherst, MA, USA; 7 Department of Radiation Oncology, University of North Carolina at Chapel Hill, Chapel Hill, NC, USA; 8 Huntsman Cancer Institute and Department of Pathology, University of Utah School of Medicine, Salt Lake City, UT, USA; 9 Department of Pathology & Laboratory Medicine, University of North Carolina at Chapel Hill, Chapel Hill, NC, USA

## Abstract

**Background:**

The epidermal growth factor receptor (EGFR/HER1) and its downstream signaling events are important for regulating cell growth and behavior in many epithelial tumors types. In breast cancer, the role of EGFR is complex and appears to vary relative to important clinical features including estrogen receptor (ER) status. To investigate EGFR-signaling using a genomics approach, several breast basal-like and luminal epithelial cell lines were examined for sensitivity to EGFR inhibitors. An EGFR-associated gene expression signature was identified in the basal-like SUM102 cell line and was used to classify a diverse set of sporadic breast tumors.

**Results:**

*In vitro*, breast basal-like cell lines were more sensitive to EGFR inhibitors compared to luminal cell lines. The basal-like tumor derived lines were also the most sensitive to carboplatin, which acted synergistically with cetuximab. An EGFR-associated signature was developed *in vitro*, evaluated on 241 primary breast tumors; three distinct clusters of genes were evident *in vivo*, two of which were predictive of poor patient outcomes. These EGFR-associated poor prognostic signatures were highly expressed in almost all basal-like tumors and many of the HER2+/ER- and Luminal B tumors.

**Conclusion:**

These results suggest that breast basal-like cell lines are sensitive to EGFR inhibitors and carboplatin, and this combination may also be synergistic. *In vivo*, the EGFR-signatures were of prognostic value, were associated with tumor subtype, and were uniquely associated with the high expression of distinct EGFR-RAS-MEK pathway genes.

## Background

The epidermal growth factor receptor (EGFR/HER1) is a member of the human epidermal growth factor receptor (HER) family of transmembrane receptor tyrosine kinases that is linked to growth control, cell adhesion, mobility, and apoptosis [1]. EGFR is an important regulator of epithelial cell biology, but its function in breast tumors is complicated by the observation that its function may vary according to important clinical features like estrogen receptor (ER) and HER2 status. Microarray studies have identified several subtypes of breast cancer arising from at least two different epithelial cell types [2-5]. Two of the molecular subtypes of breast cancer are partly defined by the high expression of ER, while a third is partly defined by the genomic DNA amplification and high expression of HER2 (i.e. HER2+/ER-, see [5]). The basal-like subtype has low expression of both ER and HER2, however, most basal-like tumors highly express EGFR as assessed by both gene and protein expression [6].

High expression of EGFR has been reported in a variety of epithelial tumors [7], leading to the development of drugs directed against this receptor [8,9]. One of these targeting strategies employs monoclonal antibodies (cetuximab) that bind the extracellular ligand-binding domain, while other strategies include small molecule inhibitors (gefitinib and erlotinib) that compete with ATP for binding to the intracellular tyrosine kinase domain [10-12]. In non-small cell lung cancer and breast cancer cell lines, it has been shown that some small molecule EGFR inhibitors increase cell killing when used in combination with chemotherapeutics [13,14]; therefore, the interactions between EGFR inhibitors and cytotoxic agents represent a promising combination for the future treatment of epithelial tumors that are dependent upon EGFR-signaling.

The lack of clinical response in breast cancers treated with gefitinib *in vivo *has been partially attributed to activation of this pathway downstream of EGFR, or ineffective methods of identifying those tumors that show an EGFR-dependent signature. EGF independent activation of the EGFR-pathway via the PI3K/AKT pathway may occur through either loss of PTEN or mutation/activation of PI3K, both of which have been linked to gefitinib resistance [15-17]. Others have suggested that the MEK/ERK pathway may play a more important role in resistance to EGFR inhibitors [18-20]. Recently, Moyano *et al*. identified αB-Crystallin (CRYAB) as a protein that can constitutively activate the MEK/ERK pathway in breast epithelial cells and caused a cell line to become EGF independent [21].

In this study, we hypothesized that the breast tumor "intrinsic" subtypes might vary in dependence upon EGFR-signaling, which could be reflective of differences in gene expression patterns. Therefore, we used breast cell lines to identify an EGFR-pathway associated profile and examined interactions between EGFR inhibitors and cytotoxic chemotherapeutics *in vitro*. These analyses identified multiple EGFR-associated profiles *in vivo *that were of prognostic significance, showed important links with tumor subtype, and highlight potential downstream activators of the EGFR-RAS-MEK pathway.

## Results

### Cell line models of breast cancer

Breast cancer is a heterogeneous disease arising from at least two distinct epithelial cell populations, therefore, we selected cell lines models of basal-like and luminal cells to begin our investigations of the EGFR-pathway. The MCF-7 and ZR-75-1 cell lines were derived from breast tumors of luminal origin and have expression of CK8/18 and ER. Our previous studies examining cell lines of basal-like origin used immortalized human mammary epithelial cell lines (HMECs) [22,23]; however, these lines are derived from normal rather than tumor tissue. Two ER-negative and HER2-non-amplified tumor-derived cell lines, SUM149 and SUM102, have been previously shown to express EGFR [18,24] and show basal-like expression profiles [25]. The SUM102 and SUM149 lines share many characteristics with the basal-like tumors including expression of CK5/6, therefore, we included these two tumor-derived lines as *in vitro *models of basal-like breast cancers. By microarray analysis, EGFR gene expression was low in the luminal cell lines and higher in the basal-like lines. EGFR protein expression by Western blot analysis was detectable in the basal-like lines, but not in the luminal lines (data not shown).

### Drug sensitivity assays

To assess EGFR inhibitor sensitivity, the six cell lines described above were treated for 72 h with a range of doses of gefitinib or cetuximab and a MTT assay was used to determine IC50 doses (Table 1). In response to gefitinib, the basal-like tumor-derived cell lines (SUM149 and SUM102) were two- to 100-fold more sensitive than the luminal lines. The two immortalized HMEC lines were 33- and 50-fold more sensitive to gefitinib than the luminal lines, suggesting that the basal-like cell lines as a whole were more sensitive to gefitinib versus the luminal cell lines. Cetuximab sensitivity was observed in only a single cell line (SUM102, IC50 = 2 ug/ml), with IC50 doses for MCF-7, ZR-75-1, SUM149, ME16C2, and HME-CC not achievable even with cetuximab doses as high as 100 ug/ml. These cell lines were also treated with inhibitors that affect targets downstream of EGFR in the pathway including U1026 (MEK1/2 inhibitor) and LY294002 (PI3K inhibitor). Most of the cell lines had a similar level of sensitivity to U0126 with the exception that SUM102 was approximately 5-fold more sensitive. IC50 doses for LY294002 were similar for most lines with the exception of ME16C and SUM149 cells, which were approximately 5-fold more resistant than the other lines. The SUM102 line was the only cell line that was sensitive to all four inhibitors and has previously been shown to be EGFR-dependent [24], and thus, was chosen for further analyses of the EGFR pathway.

**Table 1:** Estimated IC50 doses of breast cell lines treated with EGFR, MEK, and PI3K inhibitors

Cell Line	Gefitinib (μM)	Cetuximab (μg/mL)	U0126 (uM)	LY294002 (uM)
ME16C	0.3 (0.02)	>100^a^	19.7 (0.66)	21.2 (0.63)
HME-CC	0.2 (0.01)	>100^a^	12.7 (0.33)	7.3 (0.17)
SUM102	0.1 (0.002)	2.3 (0.15)	4.3 (0.20)	3.4 (0.10)
SUM149	4.7 (0.14)	>100^a^	21.8 (0.80)	18.4 (0.48)
MCF-7	21.1 (0.29)	>100^a^	17.0 (1.15)	3.9 (0.13)
ZR-75-1	11.1 (0.12)	>100^a^	25.0 (0.74)	2.4 (0.05)

72 h IC50 doses were calculated for the EGFR inhibitors gefitinib, cetuximab, the MEK1/2 inhibitor U0126, and the PI3K inhibitor LY294002.

Note that the standard errors are presented within ()

^a^No achievable IC50 dose with doses up to 100 μg/mL

### Drug combination analyses

Given the observation that most biologically targeted drugs like cetuximab typically show low response rates when tested *in vivo *alone, we examined the effects of chemotherapeutics (carboplatin, doxorubicin, 5-fluorouracil, and paclitaxel) as single agents across all cell lines and the combination of cetuximab plus chemotherapeutics in SUM102 cells. Note, we only used the SUM102 cells for the combination studies because they were the only cell line tested for which an IC50 dose for single agent cetuximab could be obtained. We also tested the combined effects of gefitinib, U0126, and LY294002 with chemotherapeutic agents in SUM102 cells. First, individual drug sensitivity (IC50 doses) for each chemotherapeutic was determined for all six cell lines (Table 2). The relative sensitivities varied across the cell lines and did not appear to correlate with cell type (i.e. basal-like vs. luminal), with the exception that the two basal-like tumor-derived cell lines (SUM102 and SUM149) were at least three-fold more sensitive to carboplatin, and at least two-fold more resistant to 5-fluorouracil when compared to their immortalized HMEC counterparts or the luminal cell lines.

**Table 2:** Estimated IC50 doses of breast cell lines treated with chemotherapeutics

Cell Line	5-Florouracil (uM)	Doxorubicin (nM)	Carboplatin (uM)	Paclitaxel (nM)
ME16C	6.0 (0.29)	32.8 (1.89)	37.5 (0.63)	0.052 (0.004)
HME-CC	1.1 (0.07)	35.5 (3.26)	48.3 (1.41)	0.025 (0.003)
SUM102	16.8 (0.82)	5.1 (0.27)	11.7 (0.26)	0.00057 (0.00001)
SUM149	28.6 (1.33)	45.0 (3.06)	7.7 (0.24)	0.71 (0.006)
MCF-7	1.2 (0.15)	56.9 (4.26)	89.4 (3.79)	0.23 (0.02)
ZR-75-1	8.4 (1.06)	26.5 (1.39)	62.6 (1.98)	0.99 (3.34)

Note that the standard errors are presented within ().

**Figure 1:**
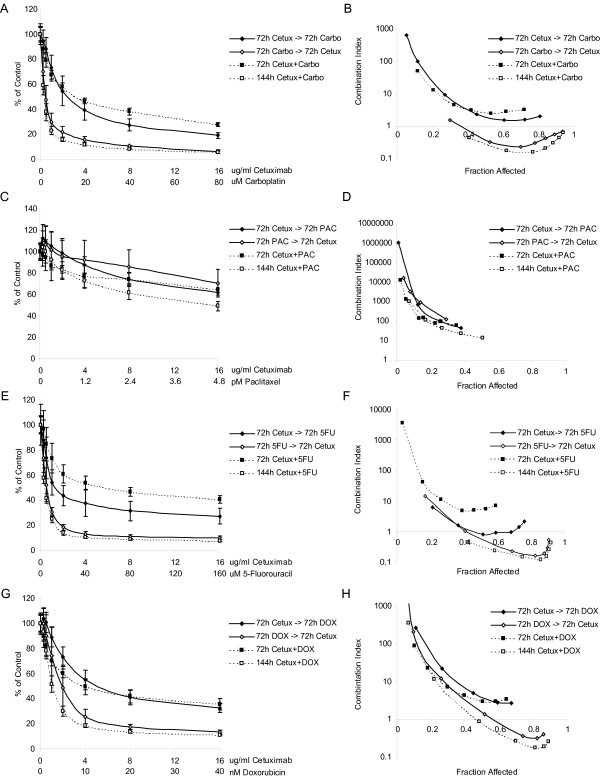
**Effects of different combination schedules of cetuximab with chemotherapeutics in SUM102 cells**. Cells were treated with four different combination schedules: 1) 72 h cetuximab followed by 72 h chemotherapy, 2) 72 h chemotherapy followed by 72 h cetuximab, 3) 72 h concurrent chemotherapy and cetuximab, and 4) 144 h concurrent chemotherapy and cetuximab. **A) **Growth inhibitory effects of cetuximab and carboplatin combinations. **B) **Combination analysis of cetuximab and carboplatin treatments. **C) **Growth inhibitory effects of cetuximab and paclitaxel combinations. **D) **Combination analysis of cetuximab and paclitaxel treatments. **E) **Growth inhibitory effects of cetuximab and 5-fluorouracil combinations. **F) **Combination analysis of cetuximab and 5-fluorouracil treatments. **G) **Growth inhibitory effects of cetuximab and doxorubicin combinations. **H) **Combination analysis of cetuximab and doxorubicin treatments. Combination Index (CI) values below one are synergistic, equal to one are additive, and greater than one are antagonistic.

As a starting point for combination experiments, we treated SUM102 cells for 72 h with cetuximab and a chemotherapeutic simultaneously. Synergistic interactions were not evident in any combination and all combinations were antagonistic as assessed by the method of Chou and Talalay in CalcuSyn [26] (Figure 1). We next analyzed the effect of sequential treatment: cells were treated for (a) 72 h with cetuximab followed by 72 h with chemotherapy, (b) 72 h with chemotherapy followed by 72 h with cetuximab, or (c) with cetuximab and chemotherapy simultaneously for 144 h. Chemotherapy followed by cetuximab was generally more growth inhibitory than cetuximab followed by chemotherapy (Figure 1). The one exception was cetuximab with paclitaxel, where all sequence combinations were antagonistic (Figure 1). However, this antagonism may result from the high sensitivity to paclitaxel already observed in the SUM102 line (Table 2). Carboplatin followed by cetuximab and the 144 h concurrent treatments were synergistic even at low doses of both drugs. 5-fluorouracil followed a similar trend to that of carboplatin, while in the doxorubicin combinations synergy was only evident at doses higher than the IC50 dose for doxorubicin first, or the 144 h concurrent (Figure 1). Similar results were observed for combinations with gefitinib and LY294002 (a PI3K inhibitor) where chemotherapy followed by each inhibitor, and the 144 h concurrent treatments, were more effective than the biological inhibitor first (data not shown). Synergy was also observed in SUM149 in addition to SUM102 for combinations of gefitinib and carboplatin (see Additional file 1). U0126 (a MEK inhibitor) combinations exhibited different results and chemotherapeutics given first followed by U0126 were slightly less synergistic than the U0126 first or concurrent treatment; however, for U0126, all combinations except doxorubicin first, or paclitaxel first, were synergistic (data not shown).

### EGFR-associated gene expression patterns *in vitro*

**Figure 2:**
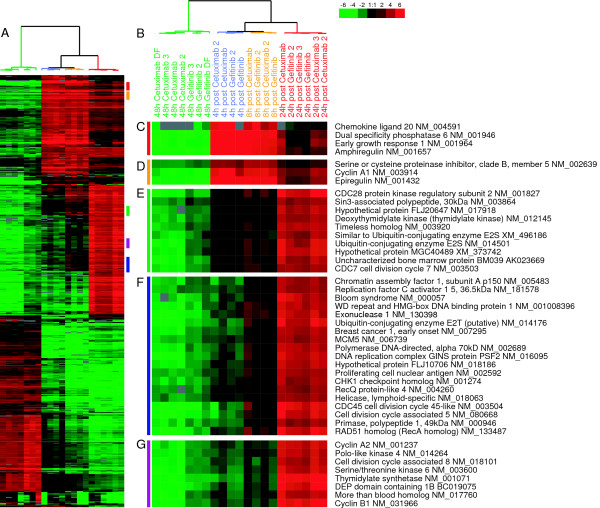
**Gene expression patterns for SUM102 cells treated with gefitinib or cetuximab**. Unsupervised hierarchical cluster analysis was performed on 48 h inhibitor treated and 4 h, 8 h, and 24 h post 48 hr inhibitor treated samples. **A) **The complete cluster overview with the colored bars indicating the location of the clusters shown in C-G. **B) **Close up of the experimental sample associated dendrogram. **C+D) **4 h and 8 h post treatment induced genes including the EGFR ligands *Amphiregulin *and *Epiregulin*. **E) **Genes involved with the G1/S phase transition induced beginning in the 4 h post inhibitor and continuing though 24 h. **F) **Genes involved in DNA synthesis induced at 8 h post inhibitor and continuing through 24 h. **G) **Proliferation genes typically observed in tumor derived profiles including *STK6 *and *Cyclin B1*.

To identify an EGFR-pathway associated profile, we analyzed the gene expression data of the SUM102 cell line treated with EGFR inhibitors (baseline) and then released from this inhibition to identify those genes that were induced upon removal of the inhibitor. Using an unsupervised analysis, we hierarchically clustered all time points from the cetuximab and gefitinib treatment experiments and identified over 500 genes that changed in expression at least 4-fold (Figure 2). Even though the two EGFR inhibitors have different mechanisms of inhibition, SUM102 cells treated for 48 h with gefitinib or cetuximab showed very similar gene expression patterns. Intra-class correlation (ICC) values between the gefitinib and cetuximab treated samples ranged from 0.627 to 0.934, and this level of similarity is evident in the short dendrogram branches from the cluster analysis (Figure 2B). The post treatment samples (i.e. after removal of inhibitor) that represent the reactivation of the EGFR-pathway were even more similar (ICC within each time point ranged from 0.862 to 0.962). A two-class SAM analysis to look for differences between gefitinib-post treatment samples versus cetuximab-post treatment samples identified only 58 significantly different genes with a false discovery rate (FDR) of 5%; thus, from a transcription standpoint, gefitinib and cetuximab elicited very similar results in SUM102 cells.

In response to gefitinib and cetuximab, the SUM102 cell line exhibited decreased expression of many proliferation genes (Figure 2). There was also a large cluster of genes that were induced by the inhibitors, consisting predominately of hypothetical genes with unknown functions. We were more interested in the genes induced after the removal of the inhibitor as this reflects the gene expression patterns associated with the reactivation of the EGFR pathway. As early as 4 h and 8 h after inhibitor removal there was a substantial increase in expression for two ligands of EGFR, namely *amphiregulin *and *epiregulin*. *Cyclin A1 *was also substantially increased (Figure 2C and 2D). Starting at 4 h and continuing through 8 h and 24 h, genes with known roles in G1/S phase such as *CDC6*, *CDC*7, *TIMELESS*, and *ORCL6 *were increased (Figure 2E and see Additional file 2). By 8 h and 24 h, DNA synthesis and DNA damage checkpoint genes were induced (Figure 2F). Classical gene expression-defined proliferation genes including *STK6 *and *Cyclin B1 *were highly induced by 24 h (Figure 2G). There was also a repression of negative regulators of growth such as *Growth arrest-specific 1 *and *Cyclin G2 *(see Additional file 2).

### Role of MEK and PI3K in the *in vitro *EGFR-profile

Activation of EGFR leads to the downstream activation of other signaling components including the MEK/ERK and PIK3/AKT pathways [1]. To examine the role of these effectors, we treated the SUM102 cell line with the MEK1/2 inhibitor U0126 and the PI3K inhibitor LY294002 alone, and in combination. Microarray time course experiments using inhibitor treated cells followed by inhibitor removal were conducted for U0126 and LY294002 similar what was done for the cetuximab and gefitinib experiments. The observed gene expression profiles for the U0126 and the LY294002 experiments were similar in both gene identity and direction when compared to the cetuximab/gefitinib profile, but gene expression changes were typically reduced in magnitude. The U0126 and LY294002 signatures when compared to each other were very similar at the 4 h and 8 h time points (average ICC = 0.83), but diverged at 24 h post treatment (average ICC = 0.59). The gene expression signatures of LY294002 and U0126 samples were also correlated with the gefitinib/cetuximab gene expression patterns at 4 h and 8 h post treatment (LY294002 compared to gefitinib/cetuximab ICC = 0.83, U0126 compared to gefitinib/cetuximab ICC = 0.77). The LY294002 and U0126 24 h post treatment samples were less correlated with gefitinib/cetuximab 24 h post samples (LY294002 compared to gefitinib/cetuximab ICC = 0.51, U0126 compared to gefitinib/cetuximab ICC = 0.41). We also treated cells with LY294002 and U0126 simultaneously to determine if the combined treatment would more completely recapitulate the EGFR-associated profile; the 24 h post combined treatment samples showed a higher correlation value to the gefitinib/cetuximab samples (average ICC = 0.73), but still did not account for the entire gene expression pattern of the 24 h post cetuximab/gefitinib treatments. These results suggest that the cetuximab/gefitinib profile could not be simply attributed to either the MEK or PIK3 pathway, but that the combination of these two pathways was more representative of the EGFR-signature than either pathway alone.

### EGFR-associated gene expression patterns *in vivo*

**Figure 3:**
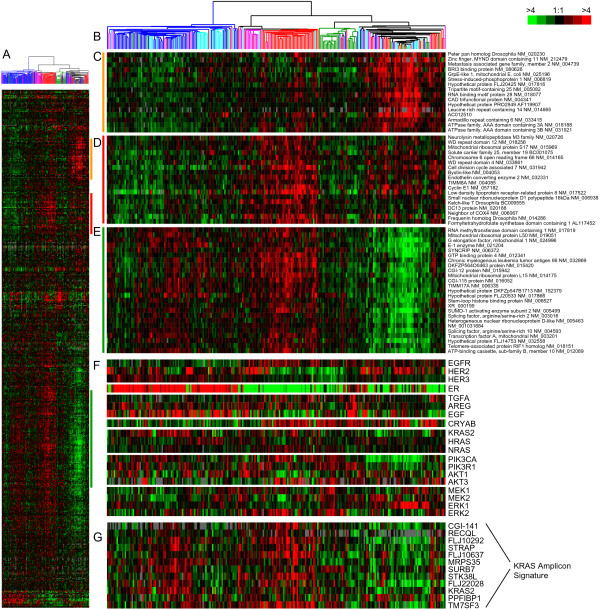
***In vivo *EGFR-associated profiles and additional genes implicated in the EGFR-RAS-MEK pathway**. **A) **The top 500 induced genes from the SUM102 post treatment experiments were hierarchical clustered using the 248 UNC tumors. Colored bars indicate the location of the three clusters in D-E. **B) **Tumor associated dendrogram color coded according to tumor subtype: Luminal A – dark blue, Luminal B – light blue, true normals and normal-like – green, HER2+/ER-negative – pink, and basal-like – red. **C) **Cluster #1 that identified a mixed group of tumors. **D) **Selected genes from the center of Cluster #2 that are high in most basal-like tumors. **E) **Selected genes from the center of Cluster #3 that are high in the luminal tumors. **F) **Data for genes with suggested roles in EGFR-pathway. **G) **Data for the *KRAS*-amplicon signature identified in Herschkowitz *et al*. [35].

To identify an EGFR-associated *in vivo *signature, a one-class SAM analysis was performed using the SUM102 cells to identify the genes that were statistically induced in the post treatment samples relative to the inhibitor treated samples. Adjusting the SAM delta value to obtain the largest gene set with less than 5% FDR resulted in a gene list that was extremely large (10,017 genes, 4.97% FDR), therefore, the top 500 induced genes were selected for further analysis (0.02% FDR). This gene list was next used to cluster 248 UNC breast tumor and normal samples representing all five breast tumor subtypes (Figure 3 and see Additional file 3). The list of induced genes from the *in vitro *SUM102 experiments were not homogenously expressed across the tumor samples; therefore, to study these multiple expression patterns in the tumors, we defined "clusters" as any gene set that contained a minimum of 20 genes and a Pearson node correlation greater than 0.55. Using this criteria, we identified three clusters: Cluster #1 was highly expressed in a mix of breast tumors that contained all five breast cancer subtypes: luminal A, luminal B, basal-like, HER2+/ER- and normal-like samples (Figure 3C, far right dendrogram branch, 35 genes); Cluster #2 identified a set of tumors that contained 58% of all basal-like tumors, 48% of all HER2+/ER- tumors and 3 luminal B tumors (Figure 3D, center dendrogram branch, 27 genes); Cluster #3 was highly enriched for luminal A and B tumors, and was also highly expressed in most of the HER2+/ER- and basal-like tumors that were also high for Cluster #2 (Figure 3E, left dendrogram branch- luminal A and B tumors, and center dendrogram branch – HER2+/ER- and basal tumors, 139 genes). Thus each gene cluster could represent a distinct EGFR-associated signature that is enriched in different subsets of tumors (for full gene lists for each cluster see Additional File 4). Gene Ontology (GO) analysis using EASE was performed on each gene cluster but only Cluster #3 had any significant GO terms, which were RNA processing, metabolism, binding, splicing, and modification (EASE scores < 0.05). *Cyclin E1 *was present within Cluster #2 and is a known prognostic marker for breast cancer patients [27]; *Cyclin E1 *is also associated with basal-like breast cancers [28,29], which was recapitulated here. Lastly, *Cyclin E1 *is known to be regulated by EGFR-signaling [30], where both AKT and ERK can inhibit p27^kip1^, which is a negative regulator of CDK2/Cyclin E1 complex [31,32].

**Figure 4:**
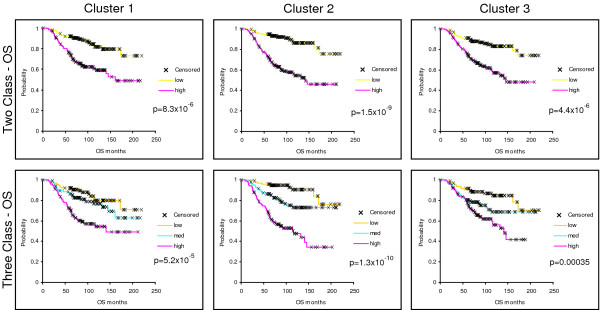
**Kaplan-Meier survival plots for the 295 NKI tumors/patients using the *in vivo *defined EGFR-associated profiles**. The average expression value for each cluster in each patient was determined and the patients then put into rank-order and divided into two equal groups or three equal groups. Overall survival analysis was performed for each cluster. X indicates censored data due loss to follow-up or to information at last checkup. Note that Clusters #2 and #3 were also similarly prognostic for the UNC 248 training data set.

To further examine the biological importance of these three EGFR-associated gene sets, we individually applied them to a test set of breast tumors (i.e. the NKI295 sample set described in [33,34]) and determine whether they predicted patient outcomes. First, we determined a mean expression value of all genes within each cluster for each patient. Next, the patients were rank-ordered according to their mean expression values for each cluster and divided into halves or thirds. Kaplan-Meier survival analyses for Relapse-Free Survival (RFS) and Overall Survival (OS) were performed and all three clusters were statistically significant predictors of outcomes where high expression always predicted a poor outcome (Figure 4 – OS; data not shown for RFS). High expression of clusters #2 and #3 were also significant predictors of RFS and OS in the UNC training data set (data not shown). Using a Cox regression analysis, we tested each cluster with the standard clinical parameters and determined that the high expression (top third) of Cluster #2 compared to the lowest expression (bottom third) significantly predicted a worse outcome for both RFS and OS (Table 3) after controlling for age, ER status, size, grade, and node status. Since the NKI295 data set was enriched for node-negative tumors less than 5 cm in diameter, tumor size and node status were not significant in the multivariate analysis [33,34]. Chi-squared analyses were performed to identify relations between tumor subtypes and Clusters #1–3. Consistent with observations from Figure 3, the basal-like, luminal B, and HER2+/ER- tumors were associated with the high expression of all three clusters while the luminal A and normal-like samples rarely showed high expression (Table 4, p =< 0.0001); in particular, the majority of basal-like tumors were almost all high for Cluster #2 (89% in top 1/3).

**Table 3:** Multivariate Cox Proportional Hazards analysis of EGFR clusters with clinical parameters in NKI295 data set

	Relapse Free Survival		Overall Survival	
Variable	Hazard Ratio (95% CI)	p-value	Hazard Ratio (95% CI)	p-value
**Standard clinical parameters**				
Age, per decade	**0.59 (0.43–0.81)**	**0.001**	**0.67 (0.45–0.99)**	**0.04**
ER status	**0.64 (0.42–0.98)**	**0.04**	**0.45 (0.27–0.71)**	**0.0009**
Size	1.38 (0.94–2.02)	0.10	1.50 (0.94–2.41)	0.09
Tumor grade 2 vs. 1	**2.41 (1.31–4.43)**	**0.005**	**4.30 (1.48–12.35)**	**0.007**
Tumor grade 3 vs. 1	**2.58 (1.38–4.81)**	**0.003**	**6.02 (2.09–17.35)**	**0.0009**
Nodes 1–3 vs. 0	0.85 (0.55–1.32)	0.48	0.91 (0.53–1.56)	0.72
Nodes >3 vs. 0	1.37 (0.83–2.26)	0.22	1.56 (0.85–2.85)	0.14
				
**Standard clinical parameters and Cluster #1**				
Age, per decade	**0.59 (0.43–0.82)**	**0.002**	**0.67 (0.45–0.99)**	**0.05**
ER status	0.67 (0.43–1.04)	0.08	**0.45 (0.27–0.75)**	**0.002**
Size	1.35 (0.92–1.99)	0.12	1.48 (0.92–2.39)	0.11
Tumor grade 2 vs. 1	**2.26 (1.23–4.18)**	**0.009**	**4.13 (1.42–11.98)**	**0.009**
Tumor grade 3 vs. 1	**2.21 (1.16–4.22)**	**0.02**	**5.34 (1.81–17.74)**	**0.002**
Nodes 1–3 vs. 0	0.82 (0.56–1.27)	0.38	0.86 (0.50–1.50)	0.60
Nodes >3 vs. 0	1.23 (0.73–2.06)	0.43	1.46 (0.79–2.71)	0.23
Cluster #1 med vs. low	1.53 (0.93–2.53)	0.10	1.25 (0.65–2.39)	0.50
Cluster #1 high vs. low	**1.70 (1.01–2.88)**	**0.05**	1.43 (0.76–2.69)	0.27
				
**Standard clinical parameters and Cluster #2**				
Age, per decade	**0.60 (0.43–0.83)**	**0.002**	**0.67 (0.452–0.99)**	**0.04**
ER status	0.73 (0.46–1.16)	0.18	**0.54 (0.32–0.91)**	**0.02**
Size	1.41 (0.96–2.07)	0.08	1.52 (0.94–2.44)	0.09
Tumor grade 2 vs. 1	**1.94 (1.05–3.61)**	**0.04**	**3.36 (1.15–9.83)**	**0.03**
Tumor grade 3 vs. 1	1.74 (0.90–3.37)	0.10	**3.54 (1.20–10.73)**	**0.02**
Nodes 1–3 vs. 0	0.80 (0.52–1.23)	0.31	0.81 (0.47–1.39)	0.44
Nodes >3 vs. 0	1.19 (0.71–1.98)	0.51	1.36 (0.74–2.49)	0.32
Cluster #2 med vs. low	**2.13 (1.22–3.71)**	**0.008**	2.10 (0.95–4.64)	0.07
Cluster #2 high vs. low	**2.63 (1.44–4.79)**	**0.002**	**3.46 (1.58–7.59)**	**0.002**
				
**Standard clinical parameters and Cluster #3**				
Age, per decade	**0.58 (0.42–0.81)**	**0.001**	**0.67 (0.45–0.98)**	**0.04**
ER status	0.68 (0.43–1.07)	0.10	**0.45 (0.27–0.76)**	**0.003**
Size	1.39 (0.95–2.03)	0.10	1.49 (0.93–2.41)	0.10
Tumor grade 2 vs. 1	**2.30 (1.24–4.24)**	**0.008**	**4.13 (1.42–12.00)**	**0.009**
Tumor grade 3 vs. 1	**2.29 (1.20–4.38)**	**0.01**	**5.38 (1.83–15.80)**	**0.002**
Nodes 1–3 vs. 0	0.83 (0.54–1.29)	0.41	0.86 (0.50–1.49)	0.60
Nodes >3 vs. 0	1.30 (0.79–2.16)	0.31	1.47 (0.80–2.70)	0.22
Cluster #3 med vs. low	1.32 (0.81–2.16)	0.26	1.54 (0.80–2.95)	0.20
Cluster #3 high vs. low	1.41 (0.84–2.37)	0.19	1.43 (0.73–2.78)	0.29

Age was a continuous variable grouped in decade years, size was a binary variable (0 = < 2 cm, 1 = > 2 cm), tumor grade 2 and 3 are relative to grade 1, and node status (1–3 nodes or > 3 nodes) was relative to 0 positive nodes. Expression of the three clusters was averaged, rank ordered, divided into equal thirds; medium and high expression is relative to low expression. Significant variables are displayed in bold.

**Table 4:** Chi-square analysis for association of gene expression with subtypes

	Basal-like	HER2+/ER-	Luminal A	Luminal B	Normal-like	p-value
# tumors	53	35	123	55	29	

Cluster 1^a^	68%	37%	12%	56%	14%	<0.0001
Cluster 2^a^	89%	49%	5%	49%	7%	<0.0001
Cluster 3^a^	77%	51%	11%	47%	0%	<0.0001
EGFR^a^	68%	20%	27%	18%	41%	<0.0001
HER2^a^	15%	100%	28%	26%	24%	<0.0001
HER4*	9%	3%	50%	38%	31%	<0.0001
TGFA^b^	74%	37%	17%	25%	38%	<0.0001
AREG^a^	3%	34%	43%	35%	41%	<0.0001
EGF	17%	40%	37%	36%	31%	0.23
CRYAB^a^	70%	11%	33%	4%	48%	<0.0001
KRAS amplicon^a^	68%	40%	24%	35%	0%	<0.0001
KRAS gene^c^	32%	37%	33%	38%	21%	0.36
HRAS^d^	32%	66%	17%	64%	7%	<0.0001
NRAS^a^	70%	28%	17%	44%	21%	<0.0001
PIK3CA	30%	17%	36%	36%	41%	0.28
PIK3R1^a^	21%	14%	42%	25%	55%	0.0012
AKT1^a^	26%	63%	27%	40%	24%	<0.0001
AKT2*	26%	40%	27%	47%	38%	0.26
AKT3^a^	51%	14%	39%	9%	45%	<0.0001
MEK1	53%	46%	25%	29%	24%	0.023
MEK2^e^	42%	43%	25%	42%	24%	0.068
ERK1^f^	30%	26%	31%	42%	41%	0.49
ERK2^g^	40%	31%	26%	45%	31%	0.048

Samples were rank ordered into three equal groups and the percentage of each subtype in the highest expression group is reported for the NKI patient data set.

*Note: HER4 could not be assessed in UNC data due to too many missing values; HER3 was not present in the NKI data set; AKT2 was not present in the UNC data set

^a ^associations were also similarly significant in the UNC sample set

^b ^nominally significant in UNC data (p-value = 0.0046)

^c ^nominally significant association in the UNC data (p-value= 0.0051)

^d ^nominally significant in the UNC data (p-value = 0.003)

^e ^nominally significant in the UNC data (p-value = 0.0023)

^f ^significant in the UNC data (p-value = 0.0003)

^g ^significant in the UNC data (p-value = <0.0001)

Bonferroni corrected level of significance α = 0.0022

### Analysis of EGFR-pathway components relative to expression patterns *in vivo*

Since most of the genes from the *in vivo *focused EGFR-associated signatures were not established members of the HER-signaling pathway, we examined the gene expression patterns of many of the known pathway components for their ability to predict patient outcomes, and determined if they showed correlations to any of the EGFR-associated profiles. Gene expression data for three-fourths of the HER family of receptors (*EGFR, HER2, HER4*), some of their ligands (*TGFA, EGF, AREG*), as well as other pathway components including *MEK1, MEK2, PIK3CA, PIK3R1, CRYAB, AKT1-3*, the *RAS *proteins (H, K and N), *ERK1*, *ERK2*, and a *KRAS*-amplicon signature (identified and defined in Herschkowitz et al. [35]), were individually tested for the ability to predict patient outcomes, for correlations with tumor subtype (Table 4), and for correlations with the EGFR-associated expression Clusters #1–3 (Table 5). Gene expression for individual genes was rank-order and divided into thirds as was done for Clusters #1–3 previously, and each gene was tested for its ability to predict outcomes in the UNC 248 and NKI 295 tumor data sets. No individual gene's expression pattern listed above significantly predicted RFS and OS in both the UNC and NKI data sets.

**Table 5:** Associations between Clusters #1–3 and individual genes using the NKI295 sample set

	Cluster 1		Cluster 2		Cluster 3	
			
	%	p-val	%	p-val	%	p-val
EGFR	39%	0.1783	43%	0.0091^b^	38%	0.15
HER2	26%	0.0017	25%	<0.0001^c^	24%	<0.0001^a^
HER4*	21%	<0.0001	12%	<0.0001	18%	<0.0001
TGFA	40%	0.0665	48%	0.0002	47%	0.0021
AREG	22%	0.0007^c^	23%	<0.0001^a^	28%	0.064^f^
EGF	35%	0.1380	25%	0.0691	27%	0.033^d^
CRYAB	35%	0.3214^f^	38%	0.0524	38%	0.0013
KRAS amplicon	38%	0.1973^e^	52%	<0.0001^c^	63%	<0.0001^a^
KRAS gene	27%	0.0022^a^	31%	0.8795	36%	0.14^e^
HRAS	48%	<0.0001^c^	51%	<0.0001	47%	0.0018
NRAS	45%	0.0362	56%	<0.0001^c^	59%	<0.0001^a^
PIK3ca	22%	0.0032^b^	27%	0.1415^e^	30%	0.33^e^
PIK3R1	24%	0.0009^a^	20%	<0.0001^a^	19%	<0.0001
AKT1	41%	0.0112	39%	0.0899	34%	0.36
AKT2*	40%	0.0519	37%	0.3524	33%	0.94
AKT3	26%	0.0004	33%	0.1569	35%	0.64^f^
MEK1	39%	0.0335	47%	0.0032^d^	48%	<0.0001
MEK2	58%	<0.0001^a^	44%	0.0113^d^	36%	0.55^f^
ERK1	37%	0.0718^e^	23%	0.0009^c^	19%	<0.0001^a^
ERK2	39%	0.0238	37%	0.3457^e^	36%	0.46^e^

Chi-squared analyses were used to identify associations between the high expression of the individual EGFR-activation profiles for each cluster (top 1/3) and the expression of individual genes categorized as high (top 1/3). The % of tumors with the high expression of each cluster and that show the high expression of the individual gene is shown.

*Note: HER4 could not be assessed in UNC data due to too many missing values; HER3 was not present in the NKI data set; AKT2 was not present in the UNC dataset.

^a ^the statistically significant association was also significant in the UNC data set (p < 0.0025).

^b ^the association was nominally significant in the NKI dataset (p < 0.05), but significant in the UNC dataset (p < 0.0025).

^c ^the association was significant in the NKI dataset (p < 0.0025), but nominally significant in the UNC dataset (p < 0.05).

^d ^the association was nominally significant in both datasets (p < 0.05).

^e ^the association was significant in UNC dataset (p < 0.0025).

^f ^the association was nominally significant in the UNC dataset (p < 0.05).

Bonferroni corrected level of significance α = 0.0025

Associations of genes or clusters with intrinsic subtype were examined using Chi-square analysis and many significant associations were identified (Tables 4). For example, high *HER2 *expression, as expected, was significantly correlated with the HER2+/ER- subtype and high *ER *expression was associated with both luminal subtypes (data not shown). *EGFR *expression was correlated with the basal-like subtype, while high *HER4*, *AREG*, and *PIK3R1 *expression was associated with the luminal A subtype. Many other associations with the basal-like subtype were also evident that included the high expression of Clusters #1–3, *TGFA, AKT3*, *CRYAB, MEK1, NRAS, KRAS *gene and the *KRAS*-amplicon signature (Table 4). Other potentially biologically relevant associations included the high expression of Clusters #2 and #3, *HRAS, MEK1*, and *AKT1 *with the HER2+/ER- subtype, and high expression of Clusters #1–3 and *HRAS *with the luminal B subtype. Even though Clusters #1–3 were identified using a basal-like tumor derived cell line, associations with luminal and HER2+/ER- tumors were identified.

We also tested for associations between the high expression of Clusters #1–3 with the high expression (i.e. top 1/3 highest group) of each of the above-mentioned genes in both the UNC and NKI datasets (Table 5). In both datasets, the high expression of *MEK2 *and *HRAS *was associated with Cluster #1, while the high expression of many other genes correlated with Clusters 2 and 3; of note was the high expression of the *KRAS*-amplicon, *HRAS, NRAS*, and *MEK1 *with both Clusters #2 and #3, and the high expression of *EGFR *with only Cluster #2. The association of different genes with the three EGFR-associated signatures is likely reflective of the complexity of signaling in this pathway across breast cancers and suggests possible driving molecular mechanisms for each EGFR-associated profile.

Lastly, a previously described mechanism for activation of the EGFR-RAS-MEK pathway is the somatic mutation of a *RAS *gene, *BRAF*, or *EGFR*, which can be relatively frequent events in non-small cell lung carcinomas. We performed sequencing analyses on a subset of the UNC breast tumors analyzed by microarray for *EGFR *mutations in exons 19 and 20, and for the common mutations in *HRAS, KRAS *and *BRAF*. No somatic sequence variants were detected in the 96 tumors that were analyzed, which were over sampled for basal-like and HER2+/ER- tumors.

## Discussion

The epidermal growth factor receptor family is of tremendous biological and clinical importance for many solid epithelial tumors. In breast cancer patients, the response rate to single agent EGFR inhibitors has been low, however, these trials were performed on unselected patient populations [36,37] and response rates might be improved within biologically selected tumor subsets. The EGFR-pathway has become a potential target in the basal-like subtype because at least 50% of basal-like tumors express EGFR as assessed by IHC [6]. Our *in vitro *analyses show that all basal-like cell lines tested were more sensitive to gefitinib compared to luminal cell lines. Only a single cell line (SUM102) was sensitive to cetuximab when EGF was present in the media, which is the condition that best mimics the *in vivo *environment [38].

Given the importance of combination therapies, we evaluated the combination of cetuximab and various chemotherapeutics in SUM102 cells and observed that the combination of cetuximab and carboplatin was highly synergistic at low doses of each drug. Even though the short-term co-treatment of cetuximab and carboplatin was antagonistic, synergism was observed in the long-term co-treatment. Carboplatin, as well as other platinum derivatives, may also be good chemotherapeutic agents for basal-like breast cancers due to the implicated function of the *BRCA1*-pathway in this subtype because *BRCA1 *mutation carriers are likely to develop tumors of the basal-like phenotype [3,39,40]. In our basal-like tumor-derived cell lines, it has been reported that the SUM149 line has a *BRCA1 *mutation and SUM102 line has barely detectable transcript levels of *BRCA1 *[41]. From a mechanistic standpoint, *BRCA1 *is required for repair of cisplatin induced DNA damage by recruiting *RAD51 *to the site of damage [42,43] and *BRCA1*-deficient cells exhibit increased sensitivity to cisplatin compared to wild type cells [44-47]. The combination of an EGFR inhibitor and a platinum drug has also been found to be synergistic in several other cell types [14,48,49]. In our experiments, we showed that not only are the basal-like tumor derived cell lines the most sensitive to carboplatin and the EGFR inhibitors when applied individually, but also that the combination was synergistic. These results provide supportive preclinical evidence for an ongoing clinical trial for "triple-negative/basal-like" (i.e. ER-negative, PR-negative, and HER2-nonamplified) metastatic breast cancer patients who are receiving either cetuximab alone versus cetuximab plus carboplatin [50].

Given the biological importance of the EGFR pathway in epithelial tumors, we identified an EGFR-associated profile *in vitro *and examined its interplay with other biological features *in vivo*. In primary breast tumors, the SUM102-defined set of EGFR-associated genes was broken into three distinct expression patterns (Figure 3), of which the high expression of two predicted poor patient outcomes in both the training and test data sets (i.e. Clusters #2 and #3). The prognostic ability of these clusters was further analyzed in the test set and Cluster #2 could predict poor outcomes even after controlling for the standard clinical parameters in a Cox multivariate analysis. Of the three signatures, Cluster #2 was the only cluster significantly associated with high EGFR gene expression.

**Figure 5:**
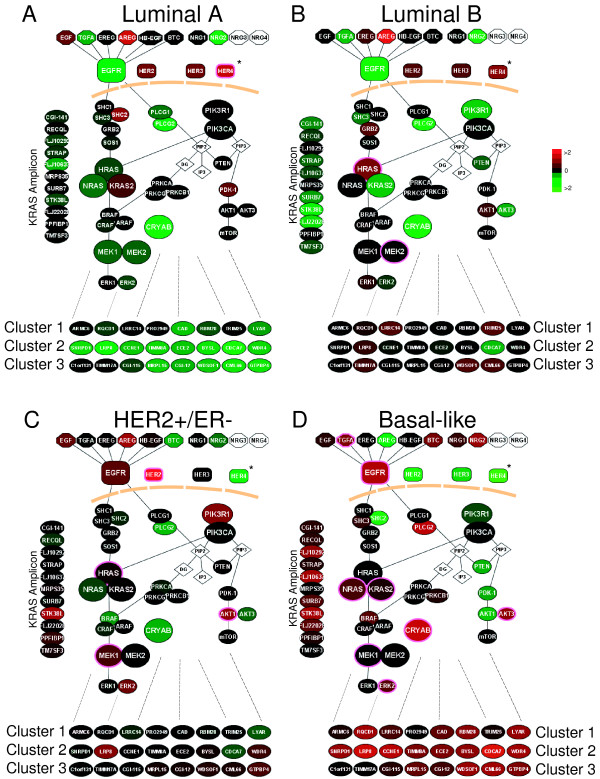
**EGFR pathway diagram displayed for each breast tumor subtype**. The average gene expression value for each gene within each subtype is displayed for the EGFR-pathway and for the three EGFR-activation profiles using the UNC 248 tumor dataset. Eight genes from the middle of each of the three EGFR-activation clusters were used to view expression of the clusters in each of the subtypes. A pink node border identifies the genes that showed statistically significant associations with subtype. *Note: the NKI HER4 data spot was used since HER4 was not present in the UNC data set. **A) **Luminal A, **B) **Luminal B, **C) **HER2+/ER- and **D) **Basal-like.

Since most of the EGFR-associated *in vivo *profile genes did not have obvious functions in the HER family pathway (aside from *Cyclin E1 *in Cluster #2), we searched for correlations with the expression levels of well known genes in the pathway. Many relationships were identified that could have important mechanistic implications (Tables 4 and 5). To assist in the interpretation of these complex patterns, we used the program Cytoscape [51,52] to display the gene expression data in a pathway styled format and highlighted the statistically significant associations observed within each subtype (Figure 5). Each subtype had a distinct EGFR-pathway cartoon relative to both the EGFR-associated profiles, as well as the expression of key genes from the EGFR-RAS-MEK pathway. The luminal A subtype showed low expression of most of the genes we examined in the HER family pathway, and on average, was low for all three EGFR-associated profiles. One of the few genes whose high expression was significantly correlated with this subtype was the *HER4 *receptor (Figure 5A); high expression of *HER4 *and average expression of two of its ligands (*HB-EGF *and *NRG1*) was observed in this tumor subtype that typically shows low grade, slow growth, and an ER-rich expression signature.

The luminal B tumors showed moderate to high expression of the EGFR-associated profiles, high *HRAS *expression, and potentially high *MEK2 *expression (Figure 5B). The EGFR-HER2 pathway has often been implicated as a potential mechanism for tamoxifen resistance in ER+ patients [36,53-57]. We determined that the high expression of the EGFR-associated profiles was able to predict outcome differences in ER+ and tamoxifen-treated patients in both the UNC and NKI data sets (data not shown); however, it should be noted that the expression of these clusters in ER+ patients closely parallels the distinction of luminal A versus luminal B. These results suggest that part of the luminal A versus luminal B distinction is due to the activation of the EGFR/HER2 pathway in luminal B tumors. In support of this hypothesis, ninety-six percent of the luminal B tumors showed high expression of at least one of the three EGFR-associated clusters, whereas only 24% of luminal A tumors had high expression of at least one. Our results are also consistent with the hypothesis of the "non-genomic" effects of ER to activate the HER pathway, where membrane bound ER complexes with EGFR and/or HER2 to cause activation of the RAS-MEK and p38 pathways [53,54,58], and suggests that these ER "non-genomic" effects are occurring in luminal B tumors. Response to EGFR inhibitors in ER-positive tumors have been mixed with some indicating a benefit [59,60], while others found no benefit [57]. A hypothesis that could be tested is that the high expression of one or more of the EGFR-associated gene sets in ER+ tumors might correlate with response/benefit to EGFR inhibitors.

The HER2+/ER- tumors, as expected, showed statistically high expression of *HER2 *and were also associated with high *HRAS *and *MEK1/MEK2 *(Figure 5C). High *AKT1 *levels were also associated with this tumor subtype, which has been previously identified [61,62].

The basal-like subtype showed the high expression of each of the three EGFR-associated profiles; ninety-one percent of the basal-like tumors had high expression of at least one of the signatures with 58% of the tumors having high expression of all three. High expression of many of the genes in the EGFR-RAS-MEK pathway were also significantly correlated with the basal-like subtype including *EGFR, TGFα, MEK1, MEK2, AKT3, CRYAB, NRAS *and the *KRAS*-amplicon signature (Figure 5D). For many of the genes or clusters examined here, as many as 70% of the basal tumors were in the highest 1/3 expression group when compared to all other tumors. These data, when coupled to the EGFR inhibitor studies on breast cells lines, strongly suggest that the EGFR-RAS-MEK pathway plays an important role in the basal-like subtype's biology, and may be a requisite activating event for tumor formation.

The pathway analysis of the basal-like subtype has also potentially provided important mechanistic clues about how the EGFR-RAS-MEK pathway is activated in basal-like tumors. One example concerns CRYAB, which has previously been shown to be highly expressed in many basal-like tumors and to portend a poor outcome. Moyano *et al*. showed that the ectopic expression of CRYAB in breast epithelial cells caused them to become transformed and EGF-independent through activation of MEK [21]. This transformed phenotype was reverted by the addition of the MEK inhibitors PD98059 and U0126, while the PIK3 inhibitor LY294002 had little effect. CRYAB could also potentially confer resistance to EGFR inhibitors as well as chemotherapy by its anti-apoptotic mechanism, which is via the inhibition of caspase-3 activation [63,64]. Other potential activation events include the high expression of *HRAS *and *KRAS*; in particular, the *KRAS*-amplicon signature (which has also been identified in a murine model of basal-like tumors[35,65]), was highly expressed in 70% of the basal-like tumors and was shown to correlate with high expression of Cluster #2. Given that most basal-like tumors showed either high expression of *CRYAB *or the *KRAS*-amplicon signature (greater than 85%), drug targeting of the EGFR-RAS-MEK pathway downstream of EGFR (i.e. MEK inhibitors) might offer a more effective therapy than targeting of EGFR directly.

While these experiments only address gene expression patterns and not the protein levels or phosphorylation status of EGFR or RAS or MEK, we believe it is likely that these signatures are *bona fide *EGFR-pathway activation signatures. The supportive data for this hypothesis includes the *in vitro *observations that these are genes induced when an EGFR-dependent cell line is freed from growth inhibition via EGFR inhibitors and the *in vivo *associations between the high expression of these signatures and genes including *HRAS*, *KRAS *and *EGFR *itself. Regardless of the classical markers of activation of the EGFR-RAS-MEK pathway, the strong associations between these expression profiles and patient outcomes in two different data sets suggest that they are important profiles. Currently, we have chosen only to validate our profiles using additional microarray data sets, as opposed to using western blots or quantitative PCR of the training set, since each of these signatures represents a large number of genes/proteins. Many of these genes have no current link to the EGFR-signaling pathway and we cannot be sure of which genes are driving the prognostic significance of the clusters. If these signatures show additional promise for clinical application, detailed follow up will dissect which genes are important for prognosis, and then they will be confirmed using other platforms. Perhaps another utility of these profiles might be the ability to predict response to EGFR inhibitors, however, we could not test this hypothesis, as there are currently no large epithelial tumor EGFR inhibitor treated microarray data sets available. However, we believe that these signatures could represent a dynamic descriptor of pathway activity compared to EGFR protein status alone, which does not predict responsiveness to EGFR inhibitors [66-68].

## Conclusion

The EGFR pathway is a complex signaling network and differences in gene expression levels of its various components can be observed across the breast cancer subtypes. EGFR-associated gene expression profiles derived *in vitro *were prognostic in two independent breast tumor data sets. Using these EGFR-associated gene expression profiles, and gene expression levels of known genes within the EGFR pathway, we have identified key differences in this pathway across the subtypes. A better understanding of each subtype's EGFR signaling pathway will have an impact on identifying and determining treatment as the gene expression signature may more readily be associated with activation of the pathway than EGFR status alone.

## Methods

### Cell culture

SUM102 and SUM149 cells were a gift from Steve Ethier of Wayne State University [69] and represent cell lines derived from ER- and HER2- basal-like breast tumors. The SUM cell lines were maintained in an Epithelial Growth Medium developed by the Tissue Culture Facility at the University of North Carolina at Chapel Hill [70], and the SUM149 line was further supplemented with 5% FBS. The MCF-7, ZR-75-1, HME-CC and ME16C cell lines were obtained and maintained as previously described [22,23].

### Cytotoxicity assay

Cell line sensitivities to drugs were assessed using a mitochondrial dye conversion assay (MTT, Cell Titer 96, Promega, Madison, WI) as described previously with the following modifications [22]. Cells were seeded into triplicate 96-well plates (SUM102, HME-CC, and ME16C – 5,000 cells/well, SUM149 – 10,000 cells/well, MCF-7 and ZR-75-1 – 7,000 cells/well) and allowed to adhere overnight. Cells were treated for 72 h with a range of doses of individual drugs. Carboplatin, doxorubicin, 5-fluorouracil, paclitaxel, and LY294002 were purchased from Sigma (St. Louis, MO). Gefitinib was a gift from AstraZeneca and cetuximab was purchased from the UNC Hospitals Pharmacy Storeroom (Chapel Hill, NC). U0126 was purchased from Cell Signaling (Danvers, MA). The inhibitory concentration that caused a 50% reduction in MTT dye conversion (IC50) dose was determined as previously described [22].

Drug combination interactions were analyzed using methods developed by Chou and Talalay [26]. Using cell lines plated as described above, seven treatment combinations consisting of constant ratios of IC50 doses (ranging from one-eighth of each dose to eight times the IC50) were applied to cells and growth compared to untreated controls using the MTT assay. Four treatment schedules were tested: 72 h concurrent, 72 h inhibitor followed by 72 h chemotherapeutic, 72 h chemotherapeutic followed by 72 h inhibitor, and a 144 h concurrent dose with a media change at 72 h (similar to the sequential treatments). CalcuSyn (BioSoft, Cambridge, UK) was used to determine the combination index, which is a measurement of the type of drug interactions. A combination index (CI) of one indicates an additive response, less than one indicates a synergistic response (greater than additive), and greater than one indicates an antagonistic response (less than additive).

### Collection of mRNA for cell line experiments

For each treatment, the SUM102 cells were grown in 15-cm dishes until 50–60% confluence. SUM102 cells were treated for 48 h with a dose equivalent to two times the 72h-IC50 dose of each inhibitor (treated samples). To identify EGFR, MEK, and PI3K activation signatures, the medium was removed after 48 h of inhibitor treatment and replaced with fresh medium without inhibitor. mRNA was harvested at 4 h, 8 h, and 24 h (post treatment samples). Cells were harvested by scraping, quickly placed into RNA lysis buffer, and mRNA was isolated using the Micro-FastTrack kit (Invitrogen, Carlsbad, CA).

### Collection of RNA for human tumor samples

248 breast tissue samples represented by 241 fresh frozen breast tumor samples and 7 normal breast tissue samples were obtained from four different sources using IRB approved protocols from each participating institution: the University of North Carolina at Chapel Hill, The University of Utah, Thomas Jefferson University and the University of Chicago; many of these samples have appeared in previous publications [71-74], and 117 are new to this study (see Additional file 5). The patients were heterogeneously treated in accordance with the standard of care dictated by their disease stage, ER, and HER2 status.

### Tumor sequence analysis

Tumor genomic DNA samples were isolated from 96 tumors using Qiagen (Valencia, CA) DNeasy Kits according to the manufacturers protocol. Gene sequencing analyses were performed at Polymorphic DNA Technologies (Alameda, CA) using an ABI 3730xl DNA sequencer and cycle sequencing, according to the manufacturers protocol. A two-step "boost/nested" PCR strategy was used where first a PCR reaction is performed to generate a larger DNA fragment, which is then used as a template for the nested reaction with a second set of PCR primers. Double stranded sequencing was performed on the nested product using the nested PCR primers as the sequencing primers. Exons 19 and 21 of *EGFR *were sequenced across all 96 patients, while exons 1 and 2 of *KRAS2*, 1 and 2 of *HRAS*, and 11 and 15 of *BRAF *were sequenced across 54 patients. No somatic alterations were detected.

### Microarray experiments

For the human tumor samples, the total RNA isolation and microarray protocols were performed as described in Hu *et al*. [5]; in this study, a number of tumor samples from previous studies were retested using a new custom Agilent microarray enriched for breast cancer genes. For cell lines experiments, labeled cRNA was generated from the mRNA using Agilent's Low RNA Input Linear Amplification Kit as described in Hu *et al*. [5]. For the cell line studies, the 48 h inhibitor treated samples were compared to an untreated cell line reference to look for effects of an inhibitor, and for the post treatment samples, to identify an activation signature for that drug/pathway. Labeled experimental sample (Cy5 CTP) and reference (Cy3 CTP) were mixed and co-hybridized overnight on the same Custom 22K Agilent Human Whole Genome Oligonucleotide Microarray described above. Two to four microarrays per experimental cell line condition were performed, including a dye-flip replicate for gefitinib- and cetuximab-treated samples. Microarrays were scanned on an Axon GenePix 4000B microarray scanner and analyzed using GenePix Pro 5.1 software. Microarray raw data were uploaded into the UNC Microarray Database and Lowess normalization was performed on the Cy3 and Cy5 channels. The microarray and patient clinical data are available at UNC Microarray Database [75] and have been deposited in the Gene Expression Omnibus under the accession number GSE6128.

### Statistical analyses

Intra-class correlations between cell line microarray experiments were performed to judge the degree of concordance between experiments/samples as described in Hu *et al*. [5]. Unsupervised analyses of the cell line samples were performed by selecting genes with an absolute signal intensity of at least 30 units (our cutoff for background signal intensity) in both channels in at least 70% of the samples tested and that also showed a Log_2 _R/G Lowess normalized ratio of two on at least two arrays. The program Cluster was used to hierarchically cluster samples and genes, and Treeview was used to view the data [76,77]. Using the SUM102 treated cells, a one-class Significance Analysis of Microarrays (SAM) was used to identify significantly induced genes in all the post treatment experiments (two to three arrays for each experimental time point) [78]. Gene ontology enrichment was assessed using EASE [79].

Analyses of the primary tumor data used the top 500 induced genes from the cell line SAM analysis described above, after filtering for 30 units in both channels in at least 70% of the tumor samples. These genes were examined in a two-way hierarchical clustering analysis with the 248 UNC tumor sample set. Three distinct expression patterns were observed and labeled as Clusters #1–3. Next, the genes in each of these three tumor-defined clusters were identified in the NKI295 patient data set [33,34], and a mean expression value for each cluster for each patient was determined. The NKI295 patients were then rank-ordered and separated into (a) two equal groups representing low and high, or (b) three equal groups representing low, medium, and high average expression for each cluster. In addition, similar gene-based rank order patient stratifications were performed for individual genes that included *EGFR*, *HER2*, *HER4*, *EGF*, *TGFA*, *AREG*, *CRYAB, KRAS, KRAS*-amplicon profile, *HRAS, NRAS, PIK3CA, PIK3R1, AKT1, AKT2, AKT3, MEK1*, *MEK2, ERK1*, and *ERK2*. Survival analyses were performed using Cox-Mantel log-rank test in Winstat for Excel (R. Fitch Software). Multivariate Cox proportional hazards analysis was performed in SAS v9.0 (SAS Statistical Software, Cary, NC) to estimate the hazard ratio associated with cluster expression in the three groups after controlling for standard clinical predictors (age, ER status, size, grade, and node status). Chi Square tests (SAS v9.0) were used to examine correlations between cluster groups, individual genes, and tumor subtype.

Gene expression relative levels were visualized in relation to the EGFR signaling pathway using Cytoscape [51,52]. The pathway was built *de novo *based on information from KEGG [80,81], BioCarta [82], and a review by Yarden and Silowkoski [1] with a focus on the RAS-MEK and PI3K/AKT components. Using the 248 UNC breast tumor microarray dataset, an average gene expression profile is displayed for the Luminal A, Luminal B, basal-like, and HER2+/ER- tumors. Tumor "intrinsic" subtype was determined for each sample using the 306 gene Centroid Predictor described in Hu *et al*. [71]; the subtype classifications used for the NKI295 sample set were also derived from this same centroid predictor and are described in Fan *et al*. [83].

## Abbreviations

EGFR/HER1: epidermal growth factor receptor; ER: estrogen receptor; HER: human epidermal growth factor receptor; MTT: mitochondrial dye conversion assay [3-(4,5-dimethylthiazol-2-yl)-2,5-diphenyltetrazolium bromide]; CI: Combination Index; SAM: Significance Analysis of Microarrays; HMEC: human mammary epithelial cell; FDR: false discovery rate; GO: gene ontology; RFS: relapse-free survival; OS: overall survival; HR: hazard ratio; 95% CI: 95% confidence interval; ICC: Intraclass correlation; PgR: progesterone receptor.

## Authors' contributions

KAH performed the cell line experiments, cell line and tumor data analysis, drafted the paper and helped with the design of the study. VJW, CF, MAT assisted with data analysis. CIS made initial observations of EGFR dependency of SUM102 cells and assisted with the discussion. Tumor sample collection, clinical data acquisition and interpretations were accomplished by LAC, LRS, TRH, and PSB. XH performed tumor RNA preparation and microarray experiments for tumor samples. CMP was the Principal Investigator, instigated and designed the study, and helped draft the paper.

## Supplementary Material

Additional file 1 Gefitinib and carboplatin combinations in breast cancer-derived cell lines. Cells were treated for 72 h with constant ratios of the IC50 doses for both gefitinib and carboplatin. Combination Index (CI) values below one are synergistic, equal to one are additive, and greater than one are antagonistic. Click here for file

Additional file 2 Full cluster diagram for the gene expression patterns of SUM102 cells treated with gefitinib or cetuximab. Click here for file

Additional file 3 Full cluster diagram for the *in vivo *EGFR-activation profiles clustered on the UNC tumor data set. Click here for file

Additional file 4 Genes from Cluster #1–3. Genes identified from the 500 SUM102 genes clustered on the UNC tumor dataset. Click here for file

Additional file 5 Clinical data associated with each tumor sample. Click here for file
