# Supplementary figures and images for: EGFR associated expression profiles vary with breast tumor subtype
Source: BMC Genomics. 2007 Jul 31;8:258. doi: 10.1186/1471-2164-8-258 (PMC2014778; doi:10.1186/1471-2164-8-258)

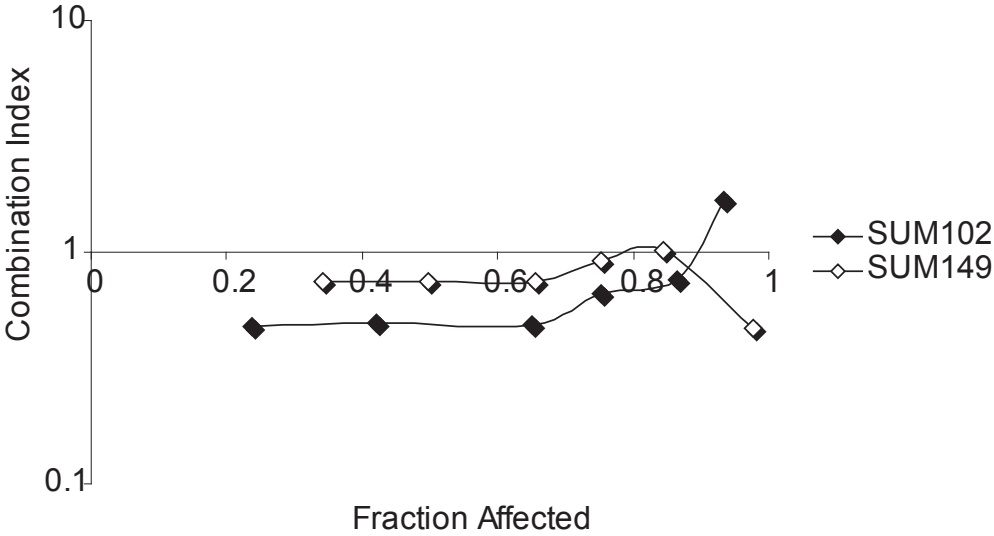

Supplement: Additional file 1 — Gefitinib and carboplatin combinations in breast cancer-derived cell lines. Cells were treated for 72 h with constant ratios of the IC50 doses for both gefitinib and carboplatin. Combination Index (CI) values below one are synergistic, equal to one are additive, and greater than one are antagonistic. [file 1471-2164-8-258-S1.pdf]

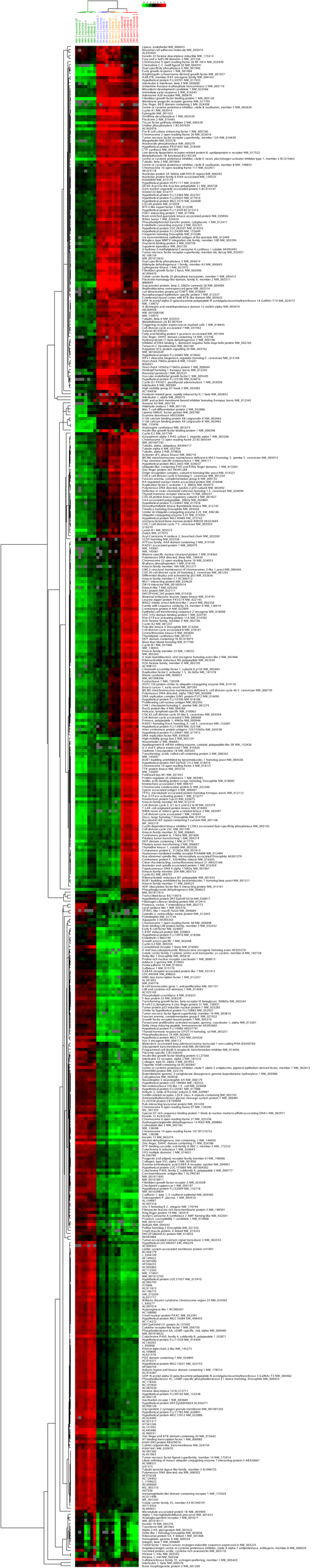

Supplement: Additional file 2 — Full cluster diagram for the gene expression patterns of SUM102 cells treated with gefitinib or cetuximab. [file 1471-2164-8-258-S2.pdf]

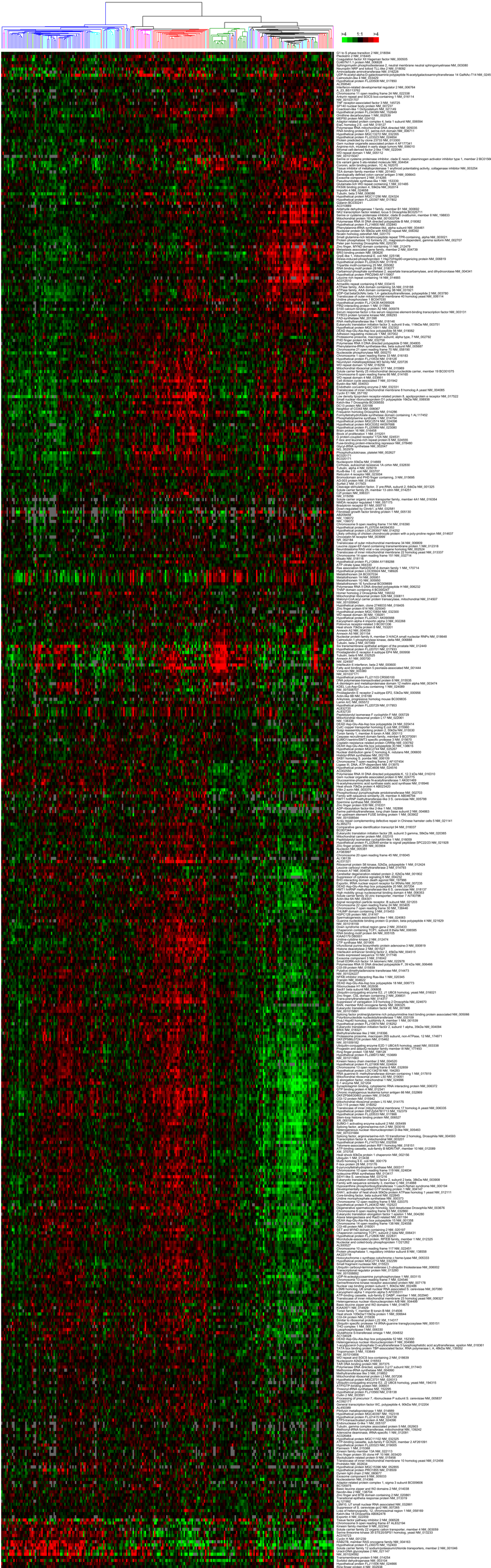

Supplement: Additional file 3 — Full cluster diagram for the in vivo EGFR-activation profiles clustered on the UNC tumor data set. [file 1471-2164-8-258-S3.pdf]
