# Supplementary material for: EGFR associated expression profiles vary with breast tumor subtype
Source: BMC Genomics. 2007 Jul 31;8:258. doi: 10.1186/1471-2164-8-258 (PMC2014778; doi:10.1186/1471-2164-8-258)
Supplement: Additional file 4 — Genes from Cluster #1–3. Genes identified from the 500 SUM102 genes clustered on the UNC tumor dataset. [file 1471-2164-8-258-S4.doc]

Supplemental Table 2. Genes from Cluster #1-3 identified from the 500 SUM102 genes clustered on the UNC tumor dataset.

| Cluster #1 | | | |
| --- | --- | --- | --- |
| Gene Symbol | Gene Name | Accession # | ClusterID |
| MGC11256 | Hypothetical protein MGC11256 | NM_024324 | Hs.211282 |
| FLJ20397 | Hypothetical protein FLJ20397 | NM_017802 | Hs.521328 |
| GAL | Galanin | BC030241 | Hs.278959 |
|  |  | AC010889 |  |
| ALDH1B1 | Aldehyde dehydrogenase 1 family, member B1 | NM_000692 | Hs.436219 |
| NKX2-5 | NK2 transcription factor related, locus 5 (Drosophila) | BC025711 | Hs.54473 |
| SERPINB8 | Serine (or cysteine) proteinase inhibitor, clade B (ovalbumin), member 8 | NM_198833 | Hs.368077 |
| MTP18 | Mitochondrial protein 18 kDa | NM_001003704 | Hs.25199 |
| POLR3B | Polymerase (RNA) III (DNA directed) polypeptide B | NM_018082 | Hs.62696 |
| FLJ14800 | Hypothetical protein FLJ14800 | NM_032840 | Hs.343334 |
| FARSLA | Phenylalanine-tRNA synthetase-like, alpha subunit | NM_004461 | Hs.23111 |
| NOL5A | Nucleolar protein 5A (56kDa with KKE/D repeat) | NM_006392 | Hs.376064 |
| NCLN | Nicalin homolog (zebrefish) | NM_020170 | Hs.501420 |
| SGTA | Small glutamine-rich tetratricopeptide repeat (TPR)-containing, alpha | NM_003021 | Hs.203910 |
| PPM1G | Protein phosphatase 1G (formerly 2C), magnesium-dependent, gamma isoform | NM_002707 | Hs.17883 |
| PPAN | Peter pan homolog (Drosophila) | NM_020230 | Hs.14468 |
| ZMYND11 | Zinc finger, MYND domain containing 11 | NM_212479 | Hs.292265 |
| MTA2 | Metastasis associated gene family, member 2 | NM_004739 | Hs.173043 |
| BRI3BP | BRI3 binding protein | NM_080626 | Hs.507227 |
| GRPEL1 | GrpE-like 1, mitochondrial (E. coli) | NM_025196 | Hs.443723 |
| STIP1 | Stress-induced-phosphoprotein 1 (Hsp70/Hsp90-organizing protein) | NM_006819 | Hs.337295 |
| LYAR | Hypothetical protein FLJ20425 | NM_017816 | Hs.425427 |
| TRIM25 | Tripartite motif-containing 25 | NM_005082 | Hs.528952 |
| RBM28 | RNA binding motif protein 28 | NM_018077 | Hs.274263 |
| CAD | Carbamoyl-phosphate synthetase 2, aspartate transcarbamylase, and dihydroorotase | NM_004341 | Hs.377010 |
| PRO2949 | Hypothetical protein PRO2949 | AF119907 | Hs.391480 |
| LRRC14 | Leucine rich repeat containing 14 | NM_014665 | Hs.459391 |
| RQCD1 | RCD1 required for cell differentiation1 homolog (S. pombe) | AC012510 |  |
| ARMC6 | Armadillo repeat containing 6 | NM_033415 | Hs.77876 |
| ATAD3A | ATPase family, AAA domain containing 3A | NM_018188 | Hs.227067 |
| ATAD3B | ATPase family, AAA domain containing 3B | NM_031921 | Hs.23413 |
| B4GALT2 | UDP-Gal:betaGlcNAc beta 1,4- galactosyltransferase, polypeptide 2 | NM_003780 | Hs.474083 |
| TOMM40 | Translocase of outer mitochondrial membrane 40 homolog (yeast) | NM_006114 | Hs.310542 |
| UPP1 | Uridine phosphorylase 1 | BC047030 | Hs.488240 |
| FLJ12438 | Hypothetical protein FLJ12438 | AK095928 | Hs.8595 |
|  |  |  |  |
| Cluster #2 | | | |
| Gene Symbol | Gene Name | Accession # | ClusterID |
| NLN | Neurolysin (metallopeptidase M3 family) | NM_020726 | Hs.247460 |
| WDR12 | WD repeat domain 12 | NM_018256 | Hs.73291 |
| MRPS17 | Mitochondrial ribosomal protein S17 | NM_015969 | Hs.44298 |
| SLC25A19 | Solute carrier family 25 (mitochondrial deoxynucleotide carrier), member 19 | BC001075 | Hs.514470 |
| C6orf66 | Chromosome 6 open reading frame 66 | NM_014165 | Hs.512144 |
| WDR4 | WD repeat domain 4 | NM_033661 | Hs.248815 |
| CDCA7 | Cell division cycle associated 7 | NM_031942 | Hs.470654 |
| BYSL | Bystin-like | NM_004053 | Hs.106880 |
| ECE2 | Endothelin converting enzyme 2 | NM_032331 | Hs.146161 |
| TIMM8A | Translocase of inner mitochondrial membrane 8 homolog A (yeast) | NM_004085 | Hs.447877 |
| CCNE1 | Cyclin E1 | NM_057182 | Hs.244723 |
| LRP8 | Low density lipoprotein receptor-related protein 8, apolipoprotein e receptor | NM_017522 | Hs.444637 |
| SNRPD1 | Small nuclear ribonucleoprotein D1 polypeptide 16kDa | NM_006938 | Hs.464734 |
| KLHL7 | Kelch-like 7 (Drosophila) | BC009555 | Hs.385861 |
| DC13 | DC13 protein | NM_020188 | Hs.388255 |
| NOC4 | Neighbor of COX4 | NM_006067 | Hs.173162 |
| FREQ | Frequenin homolog (Drosophila) | NM_014286 | Hs.301760 |
| FTHFSDC1 | Formyltetrahydrofolate synthetase domain containing 1 | AL117452 | Hs.268698 |
| PTDSS1 | Phosphatidylserine synthase 1 | NM_014754 | Hs.292579 |
| MGC2574 | Hypothetical protein MGC2574 | NM_024098 | Hs.4253 |
| MGC5352 | Hypothetical protein MGC5352 | AK097688 | Hs.102558 |
| FLJ20989 | Hypothetical protein FLJ20989 | NM_023080 | Hs.169615 |
| LOC51236 | Brain protein 16 | NM_016458 | Hs.300224 |
| BOP1 | Block of proliferation 1 | NM_015201 | Hs.535901 |
| GPR172A | G protein-coupled receptor 172A | NM_024531 | Hs.6459 |
| FBXL6 | F-box and leucine-rich repeat protein 6 | NM_024555 | Hs.12271 |
| SIAHBP1 | Fuse-binding protein-interacting repressor | NM_078480 | Hs.521924 |
|  |  |  |  |
| Cluster #3 | | | |
| Gene Symbol | Gene Name | Accession # | ClusterID |
| LCMT2 | Leucine carboxyl methyltransferase 2 | NM_014793 | Hs.200596 |
| ANXA7 | Annexin A7 | NM_004034 | Hs.386434 |
| CDR2 | Cerebellar degeneration-related protein 2, 62kDa | NM_001802 | Hs.513430 |
| SOCS6 | Suppressor of cytokine signaling 6 | NM_004232 | Hs.44439 |
| BID | BH3 interacting domain death agonist | NM_197966 | Hs.474150 |
| XPOT | Exportin, tRNA (nuclear export receptor for tRNAs) | NM_007235 | Hs.85951 |
| DDX20 | DEAD (Asp-Glu-Ala-Asp) box polypeptide 20 | NM_007204 | Hs.485810 |
| HRMT1L6 | HMT1 hnRNP methyltransferase-like 6 (S. cerevisiae) | NM_018137 | Hs.26006 |
| HMGN4 | High mobility group nucleosomal binding domain 4 | NM_006353 | Hs.236774 |
| SLC30A7 | Solute carrier family 30 (zinc transporter), member 7 | AI740796 | Hs.533903 |
| ACTL6A | Actin-like 6A | NM_004301 | Hs.435326 |
| SRPRB | Signal recognition particle receptor, B subunit | NM_021203 | Hs.12152 |
| YWHAH | Chromosome 22 open reading frame 24 | NM_003405 | Hs.226755 |
| C7orf30 | Chromosome 7 open reading frame 30 | NM_138446 | Hs.87385 |
| THUMPD3 | THUMP domain containing 3 | NM_015453 | Hs.443081 |
| HSPC128 | HSPC128 protein | NM_014167 | Hs.90527 |
| SPATA5L1 | Spermatogenesis associated 5-like 1 | NM_024063 | Hs.369657 |
| GNB4 | Guanine nucleotide binding protein (G protein), beta polypeptide 4 | NM_021629 | Hs.270543 |
|  |  | NM_001018159 |  |
| DSCR2 | Down syndrome critical region gene 2 | NM_203433 | Hs.473838 |
| CCT8 | Chaperonin containing TCP1, subunit 8 (theta) | NM_006585 | Hs.125113 |
| RBM8A | RNA binding motif protein 8A | NM_005105 | Hs.515755 |
| KIAA0179 | KIAA0179 | D80001 | Hs.129621 |
| UCK2 | Uridine-cytidine kinase 2 | NM_012474 | Hs.458360 |
| CTPS | CTP synthase | NM_001905 | Hs.473087 |
| GART | Phosphoribosylglycinamide formyltransferase, phosphoribosylglycinamide synthetase, phosphoribosylaminoimidazole synthetase | NM_000819 | Hs.473648 |
| HDAC2 | Histone deacetylase 2 | NM_001527 | Hs.3352 |
| ILF2 | Interleukin enhancer binding factor 2, 45kDa | NM_004515 | Hs.75117 |
| TEX10 | Testis expressed sequence 10 | NM_017746 | Hs.494648 |
| EXOSC3 | Exosome component 3 | NM_016042 | Hs.493887 |
| SERF1A | Small EDRK-rich factor 1A (telomeric) | NM_022978 | Hs.32567 |
| POLR3F | Polymerase (RNA) III (DNA directed) polypeptide F, 39 kDa | NM_006466 | Hs.472227 |
| CGI-09 | CGI-09 protein | NM_015939 | Hs.128791 |
| HSA9761 | Putative dimethyladenosine transferase | NM_014473 | Hs.533222 |
|  |  | NM_001024227 |  |
| NKIRAS1 | NFKB inhibitor interacting Ras-like 1 | NM_020345 | Hs.173202 |
| TSN | Translin | NM_004622 | Hs.75066 |
| DDX18 | DEAD (Asp-Glu-Ala-Asp) box polypeptide 18 | NM_006773 | Hs.363492 |
| RNASEH1 | Ribonuclease H1 | NM_002936 | Hs.502765 |
| SEC61B | Sec61 beta subunit | NM_006808 | Hs.191887 |
| UBE2J1 | Ubiquitin-conjugating enzyme E2, J1 (UBC6 homolog, yeast) | NM_016021 | Hs.163776 |
| ZCSL2 | Zinc finger, CSL domain containing 2 | NM_206831 | Hs.388087 |
| TPRT | Trans-prenyltransferase | NM_014317 | Hs.546357 |
| SUV39H2 | Suppressor of variegation 3-9 homolog 2 | NM_024670 | Hs.85567 |
| RAN | RAN, member RAS oncogene family | NM_006325 | Hs.10842 |
| EIF4E | Eukaryotic translation initiation factor 4E | NM_001968 | Hs.249718 |
|  |  | NM_001015891 |  |
| SFPQ | Splicing factor proline/glutamine rich (polypyrimidine tract binding protein associated) | NM_005066 | Hs.355934 |
| PNPT1 | Polyribonucleotide nucleotidyltransferase 1 | NM_033109 | Hs.388733 |
| DNAJA1 | DnaJ (Hsp40) homolog, subfamily A, member 1 | NM_001539 | Hs.445203 |
| FLJ10874 | Hypothetical protein FLJ10874 | NM_018252 | Hs.445386 |
| EIF2S1 | Eukaryotic translation initiation factor 2, subunit 1 alpha, 35kDa | NM_004094 | Hs.151777 |
| BRIX | BRIX | NM_018321 | Hs.38114 |
| METTL2 | Methyltransferase like 2 | NM_018396 | Hs.433213 |
| PSMD12 | Proteasome (prosome, macropain) 26S subunit, non-ATPase, 12 | NM_174871 | Hs.4295 |
| DKFZP586L0724 | DKFZP586L0724 protein | NM_015462 | Hs.463936 |
|  |  | NM_001009182 |  |
| UBE2D1 | Ubiquitin-conjugating enzyme E2D 1 (UBC4/5 homolog, yeast) | NM_003338 | Hs.129683 |
| PAQR3 | Progestin and adipoQ receptor family member III | NM_177453 | Hs.368305 |
| RNF138 | Ring finger protein 138 | NM_198128 | Hs.302408 |
| FLJ38973 | Hypothetical protein FLJ38973 | NM_153689 | Hs.471040 |
|  |  | NM_001011663 |  |
| KIF2 | Kinesin heavy chain member 2 | NM_004520 | Hs.113319 |
| FLJ21908 | Hypothetical protein FLJ21908 | NM_024604 | Hs.437855 |
| C13orf6 | Chromosome 13 open reading frame 6 | NM_032859 | Hs.183528 |
| LOC134218 | Hypothetical protein LOC134218 | NM_194283 | Hs.131887 |
| RG9MTD1 | RNA (guanine-9-) methyltransferase domain containing 1 | NM_017819 | Hs.57898 |
| MRPL50 | Mitochondrial ribosomal protein L50 | NM_019051 | Hs.288224 |
| GFM1 | G elongation factor, mitochondrial 1 | NM_024996 | Hs.518355 |
| MASA | E-1 enzyme | NM_021204 | Hs.18442 |
| SYNCRIP | Synaptotagmin binding, cytoplasmic RNA interacting protein | NM_006372 | Hs.485877 |
| GTPBP4 | GTP binding protein 4 | NM_012341 | Hs.215766 |
| CML66 | Chronic myelogenous leukemia tumor antigen 66 | NM_032869 | Hs.195870 |
| DKFZP564O0463 | DKFZP564O0463 protein | NM_015420 | Hs.532265 |
| CGI-12 | CGI-12 protein | NM_015942 | Hs.308613 |
| MRPL15 | Mitochondrial ribosomal protein L15 | NM_014175 | Hs.18349 |
| CGI-115 | CGI-115 protein | NM_016052 | Hs.408101 |
| TIMM17A | Translocase of inner mitochondrial membrane 17 homolog A (yeast) | NM_006335 | Hs.20716 |
| DKFZp547B1713 | Hypothetical protein DKFZp547B1713 | NM_152379 | Hs.434945 |
| FLJ20533 | Hypothetical protein FLJ20533 | NM_017866 | Hs.106650 |
| SLBP | Stem-loop (histone) binding protein | NM_006527 | Hs.298345 |
|  |  | XR_000199 |  |
| UBA2 | SUMO-1 activating enzyme subunit 2 | NM_005499 | Hs.511739 |
| SFRS2 | Splicing factor, arginine/serine-rich 2 | NM_003016 | Hs.73965 |
| HNRPDL | Heterogeneous nuclear ribonucleoprotein D-like | NM_005463 | Hs.527105 |
|  |  | NM_001031684 |  |
| SFRS10 | Splicing factor, arginine/serine-rich 10 (transformer 2 homolog, Drosophila) | NM_004593 | Hs.533122 |
| TFAM | Transcription factor A, mitochondrial | NM_003201 | Hs.75133 |
| FLJ14753 | Hypothetical protein FLJ14753 | NM_032558 | Hs.13453 |
| Rif1 | Telomere-associated protein RIF1 homolog | NM_018151 | Hs.536537 |
| ABCB10 | ATP-binding cassette, sub-family B (MDR/TAP), member 10 | NM_012089 | Hs.17614 |
|  |  | XM_370704 |  |
| HSPD1 | Heat shock 60kDa protein 1 (chaperonin) | NM_002156 | Hs.113684 |
| UBQLN1 | Ubiquilin 1 | NM_013438 | Hs.9589 |
| MSH6 | MutS homolog 6 (E. coli) | NM_000179 | Hs.445052 |
| FBXO28 | F-box protein 28 | NM_015176 | Hs.64691 |
| PTS | 6-pyruvoyltetrahydropterin synthase | NM_000317 | Hs.503860 |
| C10orf119 | Chromosome 10 open reading frame 119 | NM_024834 | Hs.124246 |
| IARS | Isoleucine-tRNA synthetase | NM_013417 | Hs.445403 |
| SEH1L | SEH1-like (S. cerevisiae) | NM_031216 | Hs.301048 |
| EIF2S2 | Eukaryotic translation initiation factor 2, subunit 2 beta, 38kDa | NM_003908 | Hs.429180 |
| FAM3C | Family with sequence similarity 3, member C | NM_014888 | Hs.434053 |
| HPRT1 | Hypoxanthine phosphoribosyltransferase 1 (Lesch-Nyhan syndrome) | NM_000194 | Hs.412707 |
| DRG1 | Developmentally regulated GTP binding protein 1 | NM_004147 | Hs.115242 |
| AHSA1 | AHA1, activator of heat shock 90kDa protein ATPase homolog 1 (yeast) | NM_012111 | Hs.204041 |
| CBFB | Core-binding factor, beta subunit | NM_022845 | Hs.460988 |
| UMPS | Uridine monophosphate synthetase (orotate phosphoribosyl transferase and orotidine-5'-decarboxylase) | NM_000373 | Hs.2057 |
| C12orf5 | Chromosome 12 open reading frame 5 | NM_020375 | Hs.504545 |
| FLJ40432 | Hypothetical protein FLJ40432 | NM_152523 | Hs.471234 |
| DEGS | Degenerative spermatocyte homolog, lipid desaturase (Drosophila) | NM_003676 | Hs.299878 |
| C6orf93 | Chromosome 6 open reading frame 93 | NM_032860 | Hs.185675 |
| EEF1E1 | Eukaryotic translation elongation factor 1 epsilon 1 | NM_004280 | Hs.88977 |
| ATR | Ataxia telangiectasia and Rad3 related | NM_001184 | Hs.271791 |
| DHX15 | DEAH (Asp-Glu-Ala-His) box polypeptide 15 | NM_001358 | Hs.5683 |
| C14orf138 | Chromosome 14 open reading frame 138 | NM_024558 | Hs.546431 |
| CGI-48 | CGI-48 protein | NM_016001 | Hs.463465 |
| SMYD2 | SET and MYND domain containing 2 | NM_020197 | Hs.66170 |
| CCT2 | Chaperonin containing TCP1, subunit 2 (beta) | NM_006431 | Hs.189772 |
| FLJ12806 | Hypothetical protein FLJ12806 | NM_022831 | Hs.534965 |
| MAPRE1 | Microtubule-associated protein, RP/EB family, member 1 | NM_012325 | Hs.472437 |
| NOLC1 | Nucleolar and coiled-body phosphoprotein 1 | D21262 | Hs.523238 |
|  |  | AL500527 |  |
| C10orf117 | Chromosome 10 open reading frame 117 | NM_022451 | Hs.74899 |
| PPP1R8 | Protein phosphatase 1, regulatory (inhibitor) subunit 8 | NM_138558 | Hs.533474 |
|  |  | AK223118 |  |
| HCCS | Holocytochrome c synthase (cytochrome c heme-lyase) | NM_005333 | Hs.211571 |
| MGC2714 | Hypothetical protein MGC2714 | NM_032299 | Hs.503716 |
| DKFZP566E144 | Small fragment nuclease | NM_015523 | Hs.7527 |
| UCHL3 | Ubiquitin carboxyl-terminal esterase L3 (ubiquitin thiolesterase) | NM_006002 | Hs.162241 |
| HCNGP | Transcriptional regulator protein | NM_013260 | Hs.546381 |
|  |  | NM_001008892 |  |
| UAP1 | UDP-N-acteylglucosamine pyrophosphorylase 1 | NM_003115 | Hs.492859 |
| C13orf7 | Chromosome 13 open reading frame 7 | NM_024546 | Hs.93956 |
| STRAP | Serine/threonine kinase receptor associated protein | NM_007178 | Hs.504895 |
| NCBP1 | Nuclear cap binding protein subunit 1, 80kDa | NM_002486 | Hs.522309 |
| LSM6 | LSM6 homolog, U6 small nuclear RNA associated (S. cerevisiae) | NM_007080 | Hs.190520 |
| KPNA1 | Karyopherin alpha 1 (importin alpha 5) | AF035311 | Hs.161008 |
| ABCE1 | ATP-binding cassette, sub-family E (OABP), member 1 | NM_002940 | Hs.12013 |
| TIMM23 | Translocase of inner mitochondrial membrane 23 homolog (yeast) | NM_006327 | Hs.524308 |
